# The initial impact of the SARS‐CoV‐2 pandemic on epilepsy research

**DOI:** 10.1002/epi4.12471

**Published:** 2021-02-26

**Authors:** Nancy Volkers, Samuel Wiebe, Ali Akbar Asadi‐Pooya, Ganna Balagura, Patricia Gómez‐Iglesias, Alla Guekht, Julie Hall, Akio Ikeda, Nathalie Jetté, Nirmeen A. Kishk, Peter Murphy, Emilio Perucca, Juan Carlos Pérez‐Poveda, Emmanuel O. Sanya, Eugen Trinka, Dong Zhou, J. Helen Cross

**Affiliations:** ^1^ International League Against Epilepsy Flower Mound TX USA; ^2^ Departments of Clinical Neurosciences and Community Health Sciences Cumming School of Medicine University of Calgary Calgary AB Canada; ^3^ Epilepsy Research Center Shiraz University of Medical Sciences Shiraz Iran; ^4^ Department of Neurology Jefferson Comprehensive Epilepsy Center Thomas Jefferson University Philadelphia PA USA; ^5^ Pediatric Neurology and Muscular Diseases Unit IRCCS "G. Gaslini" Institute Genova Italy; ^6^ Department of Neurosciences, Rehabilitation, Ophthalmology, Genetics, Maternal and Child Health University of Genoa Genoa Italy; ^7^ Epilepsy Unit Department of Neurology Hospital Clínico San Carlos Health Research Institute “San Carlos” (IdISCC) Universidad Complutense de Madrid Madrid Spain; ^8^ Moscow Research and Clinical Center for Neuropsychiatry Moscow Russia; ^9^ Department of Neurology, Neurosurgery and Medical Genetics Russian National Research Medical University Moscow Russia; ^10^ Department of Epilepsy, Movement Disorders and Physiology Kyoto University Graduate School of Medicine Kyoto Japan; ^11^ Division of Health Outcomes and Knowledge Translation Research Department of Neurology Icahn School of Medicine at Mount Sinai New York NY USA; ^12^ Neurology Department Faculty of Medicine Cairo University Cairo Egypt; ^13^ Epilepsy Ireland Dublin Ireland; ^14^ Division of Clinical and Experimental Pharmacology Department of Internal Medicine and Therapeutics University of Pavia, Pavia and IRCCS Mondino Foundation Pavia Italy; ^15^ ERN EpiCARE; ^16^ Neuroscience Department Faculty of Medicine Xavierian University, and Hospital Universitario San Ignacio Bogotá Colombia; ^17^ Neurology Division, Medicine Department University of Ilorin Teaching Hospital Ilorin Nigeria; ^18^ Department of Neurology Centre for Cognitive Neuroscience Christian‐Doppler University Hospital Paracelsus Medical University Salzburg Austria; ^19^ Institute of Public Health, Medical Decision‐Making and HTA UMIT Private University for Health Sciences, Medical Informatics and Technology Hall in Tyrol Austria; ^20^ Department of Neurology West China Hospital Sichuan University Chengdu China; ^21^ Programme of Developmental Neurosciences UCL NIHR BRC Great Ormond Street Institute of Child Health London UK; ^22^ Young Epilepsy Lingfield London UK; ^23^ Great Ormond Street Hospital for Children London UK

**Keywords:** COVID‐19, epilepsy care, epilepsy research, pandemic, virtual working

## Abstract

The COVID‐19 pandemic has changed the face of many practices throughout the world. Through necessity to minimize spread and provide clinical care to those with severe disease, focus has been on limiting face‐to‐face contact. Research in many areas has been put on hold. We sought to determine the impact of the COVID‐19 pandemic on epilepsy research from international basic science and clinical researchers. Responses to five questions were solicited through a convenience sample by direct email and through postings on the ILAE social media accounts and an ILAE online platform (utilizing Slack). Information was collected from 15 respondents in 11 countries by email or via Zoom interviews between May 19, 2020, and June 4, 2020. Several themes emerged including a move to virtual working, project delays with laboratory work halted and clinical work reduced, funding concerns, a worry about false data with regard to COVID research and concern about research time lost. However, a number of positive outcomes were highlighted, not least the efficiency of online working and other adaptations that could be sustained in the future.

**Key points**

- The COVID‐19 pandemic has had a major impact on epilepsy research
- The restructuring of clinical care led to delays in delivery of studies
- Funding for current and future research remains uncertain with monies diverted to COVID‐19 research and the impact on economies
- New ways of working have emerged, for example, use of digital technology, remote review, that are likely to remain in the future

## 1 INTRODUCTION

After the World Health Organization declared coronavirus disease 2019 (COVID‐19), a pandemic on March 11, 2020, social restrictions spread as quickly as the virus. Schools and universities closed. Businesses shut their doors, and people were required to practice physical distancing. Others were required to shield at home.

Healthcare services were re‐organized, which in many settings involved prioritizing the care of people affected by COVID‐19 and protecting other patients from becoming infected. Routine (nonurgent) patient care was transitioned to telehealth (eg, telephone or video visits) where feasible, with some clinicians redeployed to provide care only for COVID‐19 patients.

Medical research was dramatically impacted.^1^, ^2^, ^3^ Laboratories shut down, employing minimal staff for necessary maintenance. Most clinical studies were suspended, or processes modified to ensure the safety of participants. This resulted in delays in obtaining study data (eg, neuroimaging, neurophysiological recording) for noncritical research.

Epilepsy researchers adapted by redirecting their efforts. They tackled projects that had been on the back burner for months, or shifted to data analysis, or drafted manuscripts and grant applications. Instead of traveling to laboratories, hospitals or conferences, people commuted to home offices to watch webinars and attend virtual meetings.

After a few months, with some countries coming out of lockdown while others still under the influence of COVID‐19’s impact, more questions arise. What will the epilepsy research landscape look like going forward? Will the pandemic‐imposed changes lead to a systemic shift in how daily research is done? Has anything positive resulted from these unprecedented times?

The aim of this project was to depict a picture of the initial impacts of the COVID‐19 pandemic on epilepsy research. ILAE asked researchers in various areas of the world to share their experiences and their perceptions about what the future might hold for epilepsy research internationally.

## 2 METHODS

The five questions for the qualitative questionnaire (Table 1) were developed by the ILAE‐COVID‐19 Task Force members, with members representing each ILAE region of the world. Responses to these questions were solicited through a convenience sampling by direct email and through postings on the ILAE social media accounts and an ILAE online platform (utilizing Slack). Responses were obtained by email or Zoom interviews, depending on the respondent's preference. In addition to answering the questions, each respondent was able to add any other information they felt was relevant. Responses are identified only by the researcher's residing country.

**TABLE 1:** Questions asked of epilepsy researchers

Question
1. What research‐related changes were made at your institution due to COVID‐19?
2. Please provide one or two examples of how these changes have affected your research or your institution's research.
3. In your opinion, what might be some longer‐term effects of the pandemic on epilepsy research?
4. What is your opinion on the faster tracks for ethics approval and the increased sharing of results as preprints?
5. Please share one positive research‐related change or development that has stemmed from the pandemic.

Responses were collected and analyzed using qualitative analysis. Themes representing aspects of the initial impacts of the COVID‐19 pandemic on epilepsy research and perceptions about its expected long‐term outcomes were identified.

The study was approved by the ILAE‐COVID 19 Task Force. Ethical approval was not required. The data collected did not relate to personal health matters, other matters considered to be sensitive or confidential in nature, or matters that would be likely to damage or disturb participants or third parties. Participants were informed that the information would be publicly shared.

## 3 RESULTS

Qualitative data were obtained from 15 respondents in 11 countries between May 19, 2020, and June 4, 2020. Respondents were from Canada (2), China, Egypt, Finland, France, Germany, Italy (2), Iran, Tunisia, the United Kingdom, and the United States (3). There were 9 clinical, 4 exclusively basic science and 2 clinical with basic science. These included 11 senior/experienced, and 4 early career researchers. Table 2 outlines the data on COVID‐19 and GDP/research funding for each of the countries represented. Several themes emerged from the research questionnaires and interviews. Each theme is explored below. Excerpted quotes representing each theme are listed in Table 3.

**TABLE 2:** Metrics on COVID‐19, GDP, and Research funding within countries sampled as of the time of the interviews

Country	COVID cases as of June 15, 2020	Population	Infection rate per 100K	GDP (2019) (billions) (USD)	Per‐capita GDP (2019) (USD)	R&D as percent of GDP (2018 figures)
Canada	100 763	37 742 154	267.0	$1740	$46 195	1.57%
China	84 378	1 439 323 776	58.6	$14 340	$10 262	2.19%
Egypt	46 289	102 334 404	45.2	$303.1	$3019	0.72%
Finland	7108	5 540 720	128.3	$269.3	$48 783	2.77%
France	197 004	65 273 511	301.8	$2720	$40 494	2.20%. In 2020, public research funding was 16 billion Euro; expected to increase to 21 billion Euro in 2021 https://www.nature.com/articles/d41586‐020‐02217‐4
Germany	187 682	83 783 942	224.0	$3860	$46 445	3.09%
Iran	189 876	83 992 949	226.1	$454	$5550	0.83%
Italy	237 290	60 461 826	392.5	$2000	$33 228	1.40%
Tunisia	1110	11 818 619	9.4	$38.8	$3318	0.60%
United Kingdom	273 888	67 886 011	403.5	$2830	$42 330	1.72%
United States	2 110 000	331 002 651	637.5	$21 430	$65 298	2.84%

Note

Source for infection rates as of June 15, 2020: https://ourworldindata.org/coronavirus/country/finland?country=~FIN–cumulativecasestable

Population estimates: https://www.worldometers.info/world‐population/population‐by‐country/

GDP: https://data.worldbank.org/indicator/NY.GDP.MKTP.CD

Per‐capita GDP: https://data.worldbank.org/indicator/NY.GDP.PCAP.CD?locations=EG

Research funding as percent of GDP source: https://data.worldbank.org/indicator/GB.XPD.RSDV.GD.ZS

**TABLE 3:** Quotes illustrating themes

Theme	Representative quotes
Research projects delayed or reprioritized	“All activities that require the use of materials or consumable products have been stopped because it is impossible to obtain them, due to import restrictions.”—Tunisia “We had to stop wet lab work and no new experimental work could be started, meaning that many milestone deliverables related to grants will become delayed.”—Finland “Neuroimaging data requisition was stopped due to the quarantine, which affected our longitudinal MRI studies.”—China “Our laboratories were closed for 8 weeks, and they are now gradually re‐opening. Government regulations limit the number of people who can work simultaneously in the same laboratory.”—Italy “Many clinician investigators, including myself, were redeployed to cover internal medicine COVID teams or related services.”—USA “With decreased patient flow to the hospital, we were not able to proceed in recruiting patients [to clinical studies].”—Egypt “We were negotiating the final version of the protocol for a clinical trial. That had to be set aside, due to other priorities of the stakeholders.”—Italy
Shift to working from home & virtual meetings	“Researchers from our lab mainly worked in home offices, self‐isolating. Some stayed home due to their age or other personal risks. Some had to stay home because of their kids, which they needed to homeschool and take care of when schools and childcare centers closed.”—Germany “Face‐to face exchanges were lost between people.”—Italy “We are more efficient during [virtual] meetings and can more seamlessly share documents and knowledge together, resulting in more productive meetings.”—USA
Current and future funding concerns	“I assume some of the long‐term effects will be that a lot of money is now (and for the next couple of years) going into COVID‐19 research. That is money and attention that will be lost to other research areas, including epilepsy research.”—Germany “The huge reductions in university, charity and government income are likely to have a detrimental effect on research funding.”—UK “There's only so much funding, so by funding COVID research there will be less funding for other topics. Competition will be even more difficult.”—Canada “I think that the handling of pandemic has very clearly shown the need for fact‐based information and research. I hope the pandemic will encourage governments and funding organizations to increase research and innovation budgets.”—Finland
Effects on mental health	“Many people were struggling to adjust to the new realities. Self‐isolation or quarantine, the extremes of social distancing, no possibility to escape conflicts at home, the uncertainty… caused a lot of emotional stress and interfered with productivity.”—Germany “Mental health support services adapted for the specific needs of research or health care personnel will be critical.”—USA
Changes in the research landscape	“I am not in favor of the increasing popularity of preprints. Peer review, while imperfect, remains the best system to prevent bad science from being widely disseminated.”—Italy “The need for social distancing and infection prevention is likely to continue for months and may mean that clinical research will continue to be severely impacted.”—UK “I am concerned about the delivery of new information, as many meetings were cancelled. Though digital platforms work okay, they do not replace personal contacts and communications, during which often the most essential information is exchanged.”—Finland “It is unlikely that international travel will recover to pre‐pandemic levels in the near future. Enabling international academic exchanges without intercontinental travel is likely to be good news for the planet [from a climate change perspective].”—UK “These months of reduced interactions could break links between researchers and between centers, and that could decrease collaboration.”—Italy “The global scientific and health care community appeared to have a united front against the pandemic. This has led to multicenter collaborations for clinical trials on new drugs and the creation of databases on clinical research or medical records data from COVID‐19 patients.”—USA

### 3.1 Project delays

Basic epilepsy research was suspended in many countries with the closure of laboratories.

I have a research project on the genetics of seizures. I have been working for a couple of years to get it funded and approved. We started collecting blood samples in February 2020 … [now] it has come to a complete stop. Iran

While essential laboratory activities were allowed to continue—maintaining animal models or cell lines—all respondents stated that their university or organization had effectively shut down by mid‐March 2020, though some laboratories were cautiously reopening, with constraints, in late May 2020.

Many milestone deliverables related to grants were delayed. However, several respondents noted that these changes left them with more time to analyze a backlog of data and write manuscripts.

Researchers with children at home juggled childcare and virtual schoolwork along with their own duties. Those with teaching responsibilities also had to modify lectures for online classes and quickly adapt to university closures.

“My research was shifted toward paperwork: grant writing, paper writing, administrative tasks,” said a respondent from Germany.

Most clinical research was stalled, as trial participants could no longer visit research sites for their scheduled evaluations, including neuroimaging or biospecimen collection, except under special circumstances (eg, critical research that still required participants to come in for intravenous treatment or essential investigations). Each ongoing clinical protocol required review, as halting some studies could be harmful to participants. One researcher said that a clinical protocol on the brink of approval was set aside indefinitely, due to “competing priorities.”

Epilepsy care was affected in a myriad of ways by the pandemic. Clinical staff in hot spot areas were redeployed to care for COVID‐19 patients. Epilepsy monitoring units were closed or the rooms reallocated to COVID‐19 patients, resulting in further delays for individuals who had been waiting for weeks to months to be evaluated for epilepsy surgery. Epilepsy surgeries were also postponed, and all nonurgent procedures were deferred. Patients waiting to be evaluated for the ketogenic diet or neurostimulation devices also were left waiting, and regular follow‐up appointments were canceled or transitioned to telehealth where feasible. Emergency departments became places to avoid in many countries, leaving some families between a rock and a hard place: If their family member has a severe seizure or an epilepsy‐related adverse event, should they seek treatment or stay home?

In the greater New York City area, the early epicenter of COVID‐19 in the United States, one researcher noted that

in a world living with the daily casualties and fears of the COVID‐19 pandemic, non‐COVID‐19 related diseases that under ‘normal conditions’ were important and devastating, such as epilepsy, seemed to have dimmed into the shadows.

### 3.2 Working virtually

Although online meeting platforms and work from home arrangements have existed for years, neither was leveraged for epilepsy research—for perhaps obvious reasons. Basic science work requires a laboratory, and clinical research usually requires face‐to‐face encounters with participants and equipment and technologies not found in most homes.

The pandemic sent people home to stay—for weeks to months. Nearly every respondent,

[Virtual conferences] can provide access to high‐quality information to people who have effectively been excluded from it in the past. United Kingdom

however, mentioned virtual laboratory meetings and working from home as having also positive outcomes: They were getting a lot done, without commuting or interruption. Online meetings were often productive, thanks to seamless document sharing and better focus. Some clinical studies continued using online recruitment and assessments.

“Many of us have commented that it's a positive that people can work from home,” said a respondent from Canada. “It's much easier than we thought. It allows flexibility. It's also a lot easier to set up brief meetings, including international meetings.” “If well regulated, working at home can be much more efficient than always working on site,” said a respondent from Italy. “I hope in the future that it won't go away.”

Another respondent noted that until the pandemic, discussions by email or virtual video meeting were not accepted by the Egyptian research community. Now that the barrier has fallen, these options may accelerate and improve communication in the future.

With travel and large gatherings banned, some epilepsy conferences such as European Congress on Epileptology and Latin American Congress on Epileptology were cancelled or postponed. Others such as the European Academy of Neurology, North American Epilepsy Congress, the Eilat Conference on New Antiepileptic Drugs and Devices, and the Epilepsy Pipeline Conference pivoted to virtual formats.

Several respondents mentioned the possibility of shifting some conferences permanently to online or hybrid formats in coming years. Advantages included greater accessibility of the information and reduced environmental impact. Many noted, however, that the spontaneous conversations and discussions they valued at in‐person conferences were impossible on a virtual platform. Also, making new connections and establishing collaborations and partnerships, as often occurs during in‐person meetings, would be considered lost opportunities in virtual formats.

If all of the ‘going virtual’ continues, it can have an important positive impact on the environment, with reduced travel. United States

### 3.3 Funding concerns

Perhaps the further development and greater use of digital platforms will become more common. This would not only make high‐level international meetings available to larger audiences worldwide, but also have a positive effect on climate change, United Kingdom

Most funding organizations provided extensions and even allowed grantees to apply for additional funding to cover salaries and other expenses during the pandemic. However, all respondents expressed concerns about future funding for epilepsy research. They believed that more funding could be shunted to COVID‐19 projects and, more generally, projects on infectious diseases. Respondents also noted that economic crises in many countries would impact funding institutions, reducing grant opportunities across the board, including those for epilepsy research.

“The huge reductions in university, charity and government income are likely to have a detrimental effect on research funding,” said a respondent from the UK.

What I’m afraid of is that the economic conditions will be so bad that it will affect the amount of money going into research in general. Italy

A few respondents were optimistic, pointing out that research funding has perpetual challenges and waves of relative feast and famine and that most researchers were used to finding creative ways to fund their research and seek out new funding opportunities.

### 3.4 The changing face of research

The research world watched as institutional review boards fast‐tracked COVID‐19 clinical studies and a spate of COVID‐19 preprints (including many reporting preliminary data that would not be shared under ordinary conditions) were publicized daily as the medical community sought to understand the virus as quickly as possible. Some high impact studies as well as others have had to be retracted due to concerns about the data.^4^ The “messiness” of science was in the spotlight; some say that research emerging from the pandemic may have helped people to understand how science really works, while others worried it would undermine confidence in both research and medicine.

Most respondents indicated that fast‐tracking COVID‐19 studies and preprints were acceptable only in the context of the pandemic. “Faster science isn't better science,” said a respondent from the UK.

A respondent from Canada said,

COVID‐19 has made preprints more visible because of the importance of speed in findings, but I don’t think [that process] is applicable to standard research. If that trend happened in genetics, I would need a very high threshold for what I wanted to read, because I couldn’t read every paper and also be reviewing it at the same time.

“I believe these changes will not be sustained after the pandemic,” said a respondent from China.

The pandemic has highlighted the global importance of research and collaboration, including multicenter projects for clinical trials as well as epidemiological data collection through medical records and existing databases. There were many newly created apps, such as the COVID‐19 Symptom Study app, with nearly 4 million downloads (as of July 10—https://covid.joinzoe.com/about), which identified loss of taste and smell as a hallmark symptom of infection.^5^

The pandemic has very clearly shown the need of fact‐based information and research. I hope this will encourage governments and funding organizations to increase research and innovation budgets for bottom‐up, researcher‐initiated projects. Finland

It is difficult to imagine how the first months of the pandemic would have played out in the research world without the online infrastructure. The first months of COVID‐19 spread highlighted the need to balance big data, open access, collaborations, and speed with the deliberate pace of good research and the scientific method.

A respondent from the United States pointed out the double‐edged sword lodged in technological innovations:

While this easy to access, quick and high throughput method of producing, disseminating and analyzing data from COVID‐19 studies has been a helping hand in regulatory and scientific efforts to contain this global emergency, it came as a stark contrast with decades‐long efforts to enhance rigor of research conduct and scientific reporting and increase reproducibility and confidence in research findings.

This respondent also said that researchers have a responsibility to “preserve high levels of scientific conduct, rigor and transparency and project an accurate perception of scientific advances among both experts and the public.”

A respondent from Italy noted that conveying an accurate perception of science does not rest solely on scientists: “Media manipulation permits poor‐quality studies made available as pre‐prints or just press releases to gain extraordinary, and harmful, visibility. During the COVID‐19 pandemic, we saw a lot of that.”

Despite the months of sheltering in place and relative isolation, a respondent from Germany noted that the pandemic has brought people closer in some ways, allowing them to see sides of one another that were never possible before. A New York respondent said that “the pandemic has taught us lots of things, but the most important, in my view, is gratitude.”

### 3.5 Mental health

During the first wave of the pandemic, hospitals around the world—as well as sports stadiums and other buildings—were dedicated to COVID‐19 patient care, and some clinicians involved in epilepsy care were reassigned to care for these patients, with all the associated stress and uncertainty this entailed, including multiple daily deaths and elaborate processes for health care workers to protect themselves and their households from infection.

The physical distancing, the communication with colleagues through a mask, the minimization of face‐to face interactions, the experience of human lives lost … all are likely to leave a significant long‐lasting impact. United States (New York)

Other clinicians and researchers were homebound for weeks, where family issues could not be escaped, and videoconferences competed with domestic priorities. Some tested positive for COVID‐19 and were forced into quarantine.

During the height of the pandemic, one survey of more than 1200 physicians and nurses in China found that symptoms of depression were reported in 50% of cases, anxiety in 44%, and insomnia in 34%.^6^ Pandemic aside, clinicians are already at risk for many of these conditions, yet they are also unlikely to seek help.^7^, ^8^, ^9^

Only two respondents commented on these concerns and the potential toll on mental health. Said a respondent in New York:

The physical distancing, the communication with colleagues through a mask, the minimization of face‐to face interactions, the experience of human lives lost, whether in the hospital by health care workers or at a personal level, the discrimination into ‘high priority and COVID‐19 related’ vs ‘lower priority’ research are all likely to leave a significant long‐lasting impact. Mental health support services adapted for the specific needs of research or health care personnel will be critical.

A respondent from Germany noticed that in general, many people struggled to adjust to the isolation associated with social distancing and quarantines, as well as the impossibility of avoiding or escaping conflicts or difficulties at home.

The feeling of a permanent but invisible threat, the looks one would get if they sneezed or coughed, new duties at work and at home, and the uncertainty… all of that caused emotional distress.

The broad issues of mental health and the pandemic are reflected also in an increasing number of articles addressing mental health aspects during COVID‐19.^10^, ^11^, ^12^

### 3.6 Future challenges

Beyond the uncertainty around future funding, respondents mentioned several potential long‐term impacts from the pandemic. Long‐delayed results may become out of date. Because of shutdowns, shortages, and logistical issues in manufacturing and transportation, prices may rise for equipment and consumable products, which can affect budgets.

These months of reduced interactions could break links between researchers and between centers, and that could decrease collaboration. Italy

Challenges to clinical research could be sustained for many months, given the difficulties with face‐to‐face contact and assessments. People may be less likely to enroll in clinical trials and other research studies that involve nonurgent visits to hospitals and clinics, though telehealth may offer an option for some studies. Clinical research will have to adjust to cater for this.

Basic logistical challenges will consume time and resources. Health checks, disinfection practices, reduced occupancy, and physical distancing may limit the volume and pace of all types of epilepsy research.

In addition, the past months of isolation can never be recouped. “Time was lost,” said a respondent from Italy. “Face‐to face exchanges were lost between people. That affects science, because science is based on communication. When efficient communication is impaired, part of the research planet is gone.”

The impact of research slowdowns on patient care is also on the minds of respondents. Ultimately, a loss of time for epilepsy researchers becomes a delay in new discoveries and treatments that could improve the lives of people with epilepsy. As several respondents noted, epilepsy has not disappeared, nor will it in the future.

“At some point COVID‐19 will be gone, or at least not so relevant,” said a respondent from Canada. “But other diseases, such as epilepsy, will still be around.”

A respondent from New York agreed.

As devastating as the consequences of COVID‐19 pandemic might be, there will still be millions of people with epilepsy, hoping for a better future without seizures. The epilepsy field has made remarkable strides over the years in advancing our understanding on mechanisms, therapies, and clinical management of epilepsies as well as in advocating for people with epilepsies. More than ever, it is important to continue our efforts to advance epilepsy care and research.

## 4 DISCUSSION

The pandemic has thrown almost all aspects of our lives into sharp relief. It has forced sweeping changes over mere days. It has made people rethink their life choices and helped them discover or rediscover what is truly important to them. It has created problems but also has provoked solutions and opportunities, because if humans are anything, they are resilient.

The snapshot of opinion presented here from epilepsy researchers across the globe has highlighted the concerns as we move forward in the light of the pandemic. We acknowledge there are limitations to our methodology. We are aware that this survey involved a small sample of respondents from a limited number of countries (albeit 4 of 6 regions of the world), and therefore, results may not be representative of pandemic‐related developments in different settings. Respondents, however, included leading researchers often involved in large collaborative networks, and their feedback provided a glimpse of a range of events which affected epilepsy research in these challenging times both in their own and across other settings. We acknowledge also that there may be cross‐cultural differences. However, we wanted to have a broad opinion and felt that there would be relatively similar impact across all cultures accepting there would be differences within and without a pandemic. The views although qualitatively obtained highlight concerns about all aspects of research, not least interruption of data collection, future funding, and mental health of personnel. That aside there are also some positives to emerge, specifically with regard to models of working.

The Organisation for Economic Cooperation and Development (OECD) flash survey has garnered more than 2600 responses from nearly 100 countries (as of October 23, 2020).^13^ Most (45%) responders identify as scientists, with the rest comprising science policy advisors (20%), professionals involved in science (15%), individuals carrying out science‐related administrative work (10%), and science communicators (10%). More than 70% of respondents had shifted to working from home. Nearly 20% shifted to working on COVID‐19‐related issues. About 15% of scientists reported a reduction in the intensity of their work, while about 20% reported an increase in intensity.^13^

Having video meetings at home . . . it showed our humanity, made us laugh together, and brought us closer. People provided help to neighbors and total strangers. I hope some of this positive attitude and feeling of connectedness remains. Germany

OECD survey respondents expressed similar concerns to those of the ILAE respondents. About half expect decreases in supplies and materials, as well as funding. Half expect less job security. Most expect an increase in the use of online and digital tools for research, and a minority expects collaboration to decrease. Respondents expect science to present a stronger reputation going forward and foresee more use and integration of scientific expertise in policy advice.^13^

Respondents forecasted that some changes, such as working from home and the use of online tools for clinical research, would be sustained in some fashion. Many highlighted as a positive development the efficiencies of working from home. Although our questionnaire did not address specifically how the pandemic affected epilepsy research trainees, it is likely that the latter were similarly impacted. Compared with senior scientists, trainees may have been especially hampered by an inability to access laboratories and work at the benchside (or the bedside), resulting in delay to their training programs. It would be important for funding organizations and institutions to continue to provide support to trainees and ensure that their long‐term commitment to research remains unaffected.

However, there is widespread concern regarding the future of funding and the impacts on both human subjects’ research and patient care. Many news articles and commentaries, as well as journal articles, have explored the impacts of the pandemic on research and funding in general, and for specific conditions.^14^, ^15^, ^16^, ^17^, ^18^, ^19^, ^20^, ^21^, ^22^

In fact, governments around the world remain committed to funding research. In March 2020, the French government announced an increase of €5 billion to its science research budget; €1 billion will go toward research to prepare for future outbreaks.^23^ Peer review of grant applications has become virtual, and some funding competition deadlines were extended. The Canadian Institutes of Health Research—funded by the Canadian government—initially cancelled its spring round of grant funding in early April, after more than 2000 investigators had applied. The round was reinstated in May with a later submission deadline.^24^

As of early April, there had been no cuts to Australian government funding for health and medical research, though some funding processes had slowed. The European Research Council also is pressing forward as planned, opening a grant call with a budget of about €500 million in May. In the United States, the National Institutes of Health is maintaining research funding.

At least four nongovernmental health research foundations—the Wellcome Trust, the Gates Foundation, Howard Hughes Medical Institute, and the Chan‐Zuckerberg Initiative—are continuing to support biomedical research as planned. Major epilepsy charities, such as CURE and Epilepsy Research UK, are moving ahead with funding plans for 2020, though reduced donations may require revisions to 2021 funding forecasts. In March, the CURE Board of Directors supported funding additional grants at the close of 2020.

Major cancer charities announced budget cuts, however, which may signal future economic belt tightening across the board. In early April, Cancer Research UK, which funds about half of all UK cancer research, announced a 10% cut to its research budget and said it would postpone any new funding commitments for at least the first half of 2020. Donations had diminished dramatically, as they normally come from fundraising events and shops, neither of which were operating for several months. The American Cancer Society announced that it may be unable to fund grants at the normal level later in 2020 and that grant funding is not guaranteed for the next application cycle.^25^

Universities also anticipate declines in their income as endowments shrink under poor economic conditions, and revenue from students from abroad drops. Many hospitals have taken on pandemic‐related costs while deferring elective procedures that generate income. In the United States, some universities have announced hiring freezes, and some hospitals have cut salaries and placed employees on furlough.

Slovenia's government changed its research funding laws to allow delays in proposal evaluation, and extended deadlines for fieldwork‐dependent projects. The country's National Research Agency surveyed all research groups that receive its funding, asking if they could adapt or redirect recent proposals, if appropriate and possible, to COVID‐19‐related issues.^26^

The European Commission said it expects public and private research and development investments in the EU to drop by €3.9 billion,^27^ accounting for 1.3 per cent of the total spending foreseen for 2020. But in late May, the Commission announced plans to fund Horizon Europe with €94.4 billion in research over 7 years.^28^

The extent and breadth of impact of the COVID‐19 pandemic on epilepsy research remains to be seen as the pandemic continues and many countries enter a second wave. Any longer‐term effects on research are likely also to have impacts on patient care.

## CONFLICT OF INTEREST

EP received speaker's or consultancy fees from Amicus Therapeutics, Arvelle, Biogen, Eisai, GW Pharma, Intas Pharmaceuticals, Laboratorios Bagò, Sanofi, Sun Pharma, UCB Pharma, and Xenon Pharma. SW received unrestricted educational grants from UCB Pharma, Eisai, Livanova, and Sunovion. ET reports personal fees from EVER Pharma, Marinus, Arvelle, Medtronic, Bial—Portela & Cª, SA, NewBridge, GL Pharma, GlaxoSmithKline, Boehringer Ingelheim, Livanova, Eisai, UCB, Biogen, Genzyme Sanofi, and Actavis; his institution received grants from Biogen, UCB Pharma, Eisai, Red Bull, Merck, Bayer, the European Union, FWF Osterreichischer Fond zur Wissenschaftsforderung, Bundesministerium für Wissenschaft und Forschung, and Jubilaumsfond der Österreichischen Nationalbank outside the submitted work. AAAP receives honoraria from Cobel Daruo, RaymandRad, and Tekaje; and royalty from Oxford University Press (Book publication). AI as part of the Department of Epilepsy, Movement Disorders and Physiology as Industry‐Academia Collaboration Courses supported by Eisai, Nihon Kohden, Otsuka, and UCB Japan Co., Ltd. AI reports honorariums from Eisai, Otsuka, and UCB Japan. NJ receives an honorarium as an Associate Editor of Epilepsia. NV, GB, PGI, AG, JH, NK, PM, JCPP, ES, and DZ have no conflicts to declare. JHC has acted as an investigator for studies with GW Pharma, Zogenix, Vitaflo, and Marinius. She has been a speaker and on advisory boards for GW Pharma, Zogenix, and Nutricia; all remuneration has been paid to her department. We confirm that we have read the Journal's position on issues involved in ethical publication and affirm that this report is consistent with those guidelines.
